# Major-effect candidate genes identified in cultivated strawberry (*Fragaria* × *ananassa* Duch.) for ellagic acid deoxyhexoside and pelargonidin-3-*O*-malonylglucoside biosynthesis, key polyphenolic compounds

**DOI:** 10.1038/s41438-020-00347-4

**Published:** 2020-08-01

**Authors:** Jahn Davik, Kjersti Aaby, Matteo Buti, Muath Alsheikh, Nada Šurbanovski, Stefan Martens, Dag Røen, Daniel James Sargent

**Affiliations:** 1 Division of Biotechnology and Plant Health, Norwegian Institute of Bioeconomy Research, Ås, N-1433 Norway; 2 NOFIMA AS, Norwegian Institute of Food Fisheries and Aquaculture Research, Ås, N-1433 Norway; 3 Department of Agriculture, Food, Environment and Forestry, University of Florence, Florence, Italy; 4 Graminor Breeding Ltd., N-2322 Ridabu, Norway; 5 Department of Plant Sciences, Norwegian University of Life Sciences, Ridabu, N-1432 Ås Norway; 6 NIAB-EMR, East Malling, ME19 6BJ Kent, UK; 7 Department of Food Quality and Nutrition, Fondazione Edmund Mach, Centro Ricerca e Innovazione, Via E. Mach 1, 38010 San Michele all’Adige, TN Italy; 8 Department of Genetics, Genomics and Breeding, NIAB-EMR, East Malling, ME19 6BJ Kent, UK

**Keywords:** Genetic association study, Plant genetics, Genetic markers

## Abstract

Strawberries are rich in polyphenols which impart health benefits when metabolized by the gut microbiome, including anti-inflammatory, neuroprotective, and antiproliferative effects. In addition, polyphenolic anthocyanins contribute to the attractive color of strawberry fruits. However, the genetic basis of polyphenol biosynthesis has not been extensively studied in strawberry. In this investigation, ripe fruits from three cultivated strawberry populations were characterized for polyphenol content using HPLC-DAD-MS^n^ and genotyped using the iStraw35k array. GWAS and QTL analyses identified genetic loci controlling polyphenol biosynthesis. QTL were identified on four chromosomes for pelargonidin-3-*O*-malonylglucoside, pelargonidin-3-*O*-acetylglucoside, cinnamoyl glucose, and ellagic acid deoxyhexoside biosynthesis. Presence/absence of ellagic acid deoxyhexoside and pelargonidin-3-*O*-malonylglucoside was found to be under the control of major gene loci on LG1X2 and LG6b, respectively, on the *F.* × *ananassa* linkage maps. Interrogation of gene predictions in the *F. vesca* reference genome sequence identified a single candidate gene for ellagic acid deoxyhexoside biosynthesis, while seven malonyltransferase genes were identified as candidates for pelargonidin-3-*O*-malonylglucoside biosynthesis. Homologous malonyltransferase genes were identified in the *F.* × *ananassa* ‘Camarosa’ genome sequence but the candidate for ellagic acid deoxyhexoside biosynthesis was absent from the ‘Camarosa’ sequence. This study demonstrated that polyphenol biosynthesis in strawberry is, in some cases, under simple genetic control, supporting previous observations of the presence or absence of these compounds in strawberry fruits. It has also shed light on the mechanisms controlling polyphenol biosynthesis and enhanced the knowledge of these biosynthesis pathways in strawberry. The above findings will facilitate breeding for strawberries enriched in compounds with beneficial health effects.

## Introduction

Commercial production of the cultivated strawberry (*Fragaria* × *ananassa* Duch.) has increased steadily in recent years with ~12.9 million tons of fruit sold globally in 2017 (http://www.fao.org/faostat/). Increased consumer demand for strawberries is partly due to a greater health consciousness among the consumers and an awareness of the health promoting benefits associated with the consumption of fresh fruits. Strawberries have been shown to contain a wealth of ‘health promoting’ compounds, many of which have been reported to play a role in reducing risk factors for cardiovascular diseases^1,2^. Strawberries are rich in dietary fiber, vitamin C and a range of secondary plant metabolites, including polyphenol compounds which exert numerous positive health benefits to consumers^3–5^. Ingested polyphenols are predominantly utilized in the colon, where the gut microbiota converts isoflavones, ellagitannins, and lignans to equol, urolithins, and enterolignans, respectively, which have anti-inflammatory effects and induce antiproliferative activities in humans^4^. Recent studies have also shown that bioactive metabolites derived from dietary polyphenols by gut microbiota exert neuroprotective effects upon crossing the blood–brain barrier^6^ and polyphenols have been evaluated as therapeutics for neurodegenerative diseases^3^.

From the perspective of breeding, polyphenolic compounds have received attention not only because of their beneficial health effects, but also because they contribute to enhanced sensorial properties of the berry experience for the consumer^1,7,8^. Indeed, strawberries are one of the fruits richest in ellagitannins which together with the anthocyanins and proanthocyanidins, represent the highest proportion of their polyphenol content^9–12^. Polyphenol compounds that accumulate in ripe strawberries include flavonoids, comprising anthocyanins, flavonols and flavan-3-ols, as well as phenolic acids and ellagitannins^10,11^. The anthocyanins, which are responsible for the red color of the berries, consist of four main compounds; pelargonidin-3-*O*-glucoside, pelargonidin-3-*O*-malonylglucoside and, to a lesser extent, pelargonidin-3-*O*-rutinoside, and cyanidin-3-*O*-glucoside^10,12^. The genetic control of anthocyanin biosynthesis has been extensively studied in the diploid strawberry species *F. vesca*. Initially, a single genetic locus (*c*) was shown to be responsible for the yellow-fruited mutants of the species such as ‘Yellow Wonder’^13,14^ and the gene *Flavanone-3-hydroylase* (*F3H*) in the anthocyanin biosynthesis pathway was proposed as a candidate gene for this locus^15^. Subsequently, however, candidate SNPs in the transcription factor *FveMYB10* were confirmed to be responsible for the yellow-fruited *F. vesca* mutants^16^. The main flavonols present in strawberries are glycosides and glucuronides of kaempferol and quercetin. The phenolic acids are predominantly cinnamic acid derivatives, while the most abundant ellagitannin in strawberry is agrimoniin^10^. Although the phenolic composition of ripe strawberries has been shown to vary considerably between cultivars^10–12^ and was demonstrated to be under strong genetic control in diploid *Fragaria* species^17^, knowledge of the genetic basis of polyphenol biosynthesis and accumulation in strawberry remains scarce.

In order to breed for higher concentrations of health-related phytochemicals such as polyphenols in the cultivated strawberry, it is first essential to understand the inheritance of such compounds. Many quantitative trait loci (QTL) mapping studies have been undertaken to investigate various aspects of cultivated strawberry fruit quality, including the identification of QTL for total anthocyanin content^18^, overall sweetness, titratable acidity and ascorbic acid content^19^, and fruit primary metabolite content^20^. More recently, a study of a ‘Delmarvel’ × ‘Selva’ progeny identified many QTL similar to previous studies^21^, and QTL for fruit quality have been identified in 23 related F_1_ progenies using a pedigree-based approach^22^.

While numerous reported studies have characterized the polyphenolic content of the ripe fruit of a diverse array of strawberry cultivars^9,11,12,23^, the only investigation to date that has precisely characterized the ripe fruit content of individual polyphenolic compounds in a segregating mapping population was that of Urrutia et al.^17^ in diploid *Fragaria*. In that study, the authors determined the polyphenol content of the fruits of a wild diploid NIL progeny using LC–ESI–MS^n^ and reported 76 stable QTL for the genetic control of 22 distinct polyphenolic compounds including anthocyanins, flavonols, flavan-3-ols, flavanones, hydroxycinnamic acid derivatives, and ellagitannins. However, for the most part, the QTL intervals defined in that study were large, spanning considerable physical distances on the diploid *F. vesca* genome.

The aim of this investigation was to study the inheritance of genes controlling polyphenol biosynthesis in the cultivated strawberry by characterizing the ripe fruits of three mapping populations from parental lines that had previously been shown to differ in the polyphenol content of their berries^10^ using HPLC-DAD-MS^n^ against known standards. The progenies of the three mapping populations were genotyped, and GWAS and QTL analyses were performed. Following the identification of resistance QTL and genetic loci of major effect, the diploid *F. vesca* and octoploid cultivated strawberry reference genome were mined for candidate genes.

## Results

### Characterization of polyphenolic compounds in parental and progeny lines

**Fig. 1:**
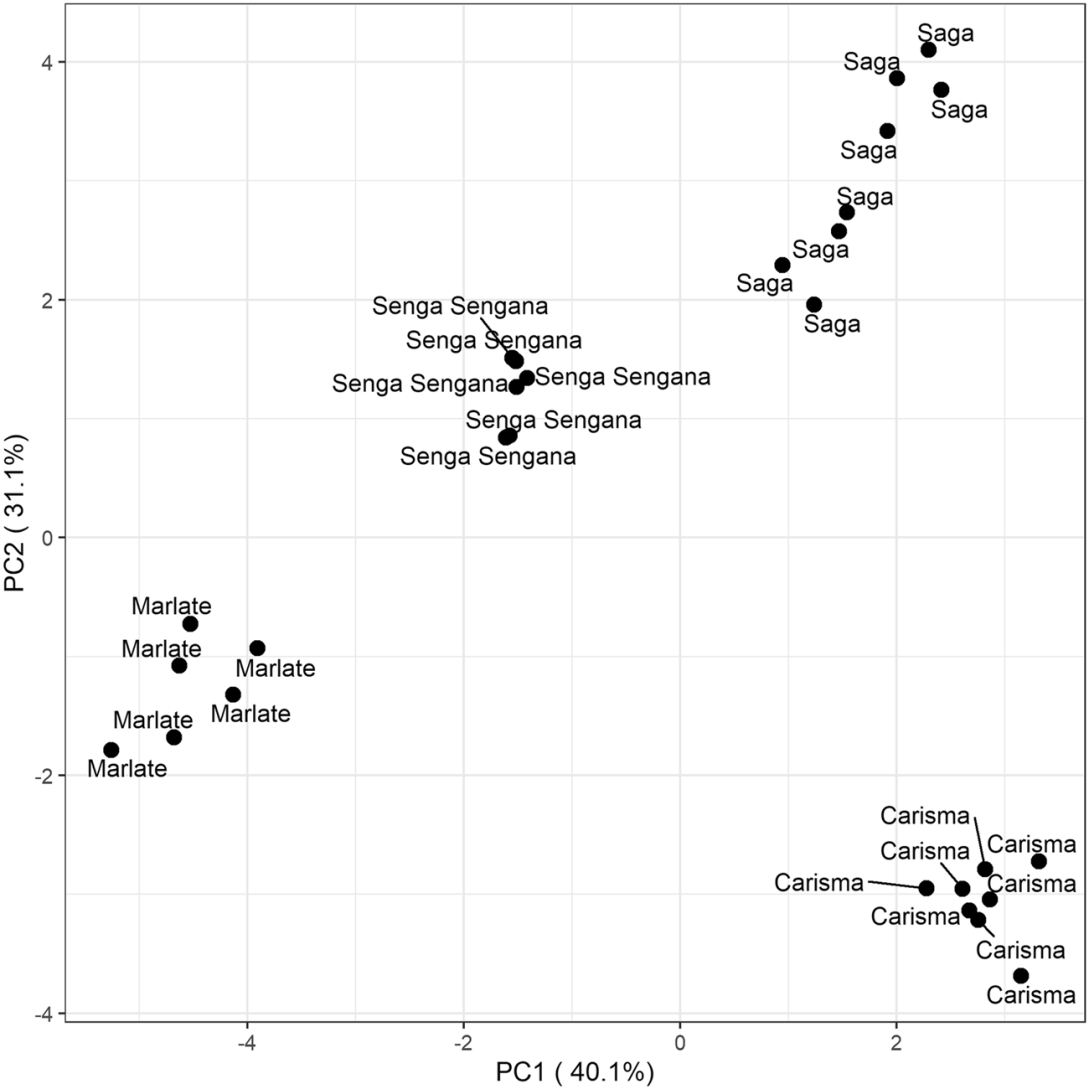
Principal components plot of total polyphenol composition of the four cultivars used as parents of the mapping populations investigated. The scores of the two first principal components are plotted using standardized polyphenol concentrations of the cultivars as input

The mean fruit concentrations for six anthocyanins, five cinnamic acids, four ellagic acid derivatives, one ellagitannin (agrimoniin), and five flavonols, are presented in Table 1. For the parents, standard errors are presented together with the marginal means to indicate the precision of the measurements, while for the F_1_ hybrids, standard deviations are given to demonstrate the spread of the observations in each progeny^24^. A principal components plot derived from all polyphenolic compounds in the four parental cultivars (Table 1) is given in Fig. 1.

**Table 1:** Concentrations (mg 100 g^−1^ of FW) of phenolic compounds analyzed in ripe fruit samples in the parental genotypes of the three mapping populations studied, along with the mean concentrations observed in the progeny of each mapping population

Polyphenol:	‘Carisma’	‘Marlate’	‘Saga’	‘Senga Sengana’	All F_1_-hybrids	‘Carisma’ × ‘Senga Sengana’	‘Marlate’ × ‘Senga Sengana’	‘Saga × ‘Senga Sengana’
Anthocyanins:								
Cyanidin-3-*O*-glucoside	1.000 ± 0.033	0.083 ± 0.054	1.463 ± 0.089	1.517 ± 0.185	1.340 ± 0.955	1.484 ± 0.809	0.580 ± 0.325	1.993 ± 1.110
Pelargonidin-3-*O*-glucoside	26.27 ± 1.01	11.43 ± 1.15	32.44 ± 2.75	25.10 ± 0.73	26.56 ± 10.45	29.41 ± 9.01	19.49 ± 8.96	30.91 ± 9.54
Pelargonidin-3-*O*-rutinoside	2.238 ± 0.050	0.0 ± 0.0	5.175 ± 0.349	1.367 ± 0.033	1.97 ± 1.699	3.492 ± 1.453	0.288 ± 0.440	2.102 ± 1.044
Cyanidin-3-*O*-malonylglucoside	0.0 ± 0.0	0.0 ± 0.0	0.0 ± 0.0	0.100 ± 0.063	0.107 ± 0.216	0.038 ± 0.099	0.024 ± 0.096	0.267 ± 0.298
Pelargonidin-3-*O*-malonylglucoside	0.0 ± 0.0	2.783 ± 0.378	5.275 ± 0.597	4.050 ± 0.134	3.358 ± 2.946	1.935 ± 1.907	2.811 ± 1.792	5.447 ± 3.613
Pelargonidin-3-*O*-acetylglucoside	0.013 ± 0.013	0.0 ± 0.0	0.238 ± 0.026	0.033 ± 0.021	0.133 ± 0.213	0.153 ± 0.182	0.040 ± 0.098	0.207 ± 0.287
Total anthocyanins	29.54 ± 1.07	14.30 ± 1.58	44.63 ± 3.78	32.10 ± 1.14	33.50 ± 13.73	36.55 ± 11.05	23.23 ± 11.04	40.97 ± 12.45
Cinnamic acids:								
Cinnamoyl glucose	4.363 ± 0.195	28.933 ± 2.922	7.413 ± 0.529	10.58 ± 0.992	13.078 ± 9.822	8.752 ± 5.748	20.369 ± 12.145	10.078 ± 5.252
*p*-Caffeoylhexose	0.213 ± 0.139	0.233 ± 0.148	0.0 ± 0.0	0.217 ± 0.138	0.725 ± 0.658	0.600 ± 0.725	1.068 ± 0.513	0.502 ± 0.573
*p*-Coumaroylhexose 1	10.25 ± 0.39	6.00 ± 0.208	4.70 ± 0.285	4.85 ± 0.397	6.627 ± 3.656	8.207 ± 4.202	5.570 ± 2.949	6.046 ± 3.121
*p*-Coumaroylhexose 2	1.613 ± 0.061	0.817 ± 0.031	0.0 ± 0.0	0.983 ± 0.040	0.843 ± 0.577	1.044 ± 0.640	0.812 ± 0.534	0.661 ± 0.480
Ferulic acid hexose derivative	1.138 ± 0.065	0.0 ± 0.0	0.763 ± 0.132	1.183 ± 0.048	0.621 ± 0.579	0.841 ± 0.527	0.249 ± 0.429	0.776 ± 0.581
Total cinnamic acids	17.55 ± 0.76	35.93 ± 3.15	12.88 ± 0.50	17.80 ± 1.46	21.90 ± 11.32	19.44 ± 9.05	28.07 ± 13.37	18.07 ± 8.12
Ellagic acids:								
Ellagic acid pentoside	0.250 ± 0.094	0.650 ± 0.056	0.00 ± 0.0	0.417 ± 0.031	0.261 ± 0.299	0.093 ± 0.219	0.510 ± 0.270	0.180 ± 0.224
Ellagic acid deoxyhexoside	0.938 ± 0.038	0.0 ± 0.0	0.525 ± 0.053	0.0 ± 0.0	0.305 ± 0.315	0.620 ± 0.189	0.002 ± 0.015	0.284 ± 0.263
Ellagic acid	0.538 ± 0.038	0.617 ± 0.060	0.263 ± 0.060	0.317 ± 0.079	0.354 ± 0.193	0.363 ± 0.173	0.413 ± 0.168	0.282 ± 0.215
Total ellagic acids	1.675 ± 0.080	1.267 ± 0.106	0.788 ± 0.117	0.733 ± 0.109	0.916 ± 0.363	1.076 ± 0.393	0.925 ± 0.338	0.746 ± 0.278
Ellagitannin								
Agrimoniin	9.250 ± 0.163	4.167 ± 0.191	6.338 ± 0.450	2.917 ± 0.149	4.677 ± 1.871	5.388 ± 1.999	3.550 ± 1.482	5.096 ± 1.532
Flavonols:								
Quercetin-3-*O*-glucuronide	3.463 ± 0.151	0.733 ± 0.042	3.500 ± 0.216	1.700 ± 0.193	2.475 ± 1.317	3.227 ± 1.359	1.302 ± 0.601	2.898 ± 0.921
Quercetin-3-*O*-malonylglucoside	0.013 ± 0.013	0.183 ± 0.017	0.050 ± 0.019	0.133 ± 0.021	0.116 ± 0.151	0.057 ± 0.079	0.115 ± 0.120	0.181 ± 0.204
Kaempferol-3-*O*-glucuronide	1.425 ± 0.037	0.250 ± 0.022	0.563 ± 0.026	0.267 ± 0.021	0.526 ± 0.371	0.916 ± 0.294	0.247 ± 0.166	0.403 ± 0.223
Kaempferol-3-*O*-malonylglucoside	0.0 ± 0.0	0.283 ± 0.022	0.263 ± 0.031	0.200 ± 0.0	0.220 ± 0.178	0.152 ± 0.145	0.254 ± 0.161	0.258 ± 0.206
Kaempferol-3-*O*-coumaroylglucoside	0.150 ± 0.019	0.200 ± 0.026	0.425 ± 0.031	0.433 ± 0.021	0.320 ± 0.163	0.288 ± 0.188	0.306 ± 0.129	0.369 ± 0.157
Total flavonols:	5.075 ± 0.191	1.683 ± 0.091	4.813 ± 0.159	2.733 ± 0.214	3.665 ± 1.600	4.654 ± 1.489	2.233 ± 0.851	4.107 ± 1.220

For the parental lines, the standard error of the means is given, while for the hybrid populations the standard deviation is presented. Values for parents are mean ± 1 SE (*n* = 6–8). For hybrids the values are mean ± 1 SD (*n* = 2)

### Genome-wide association analysis

**Fig. 2:**
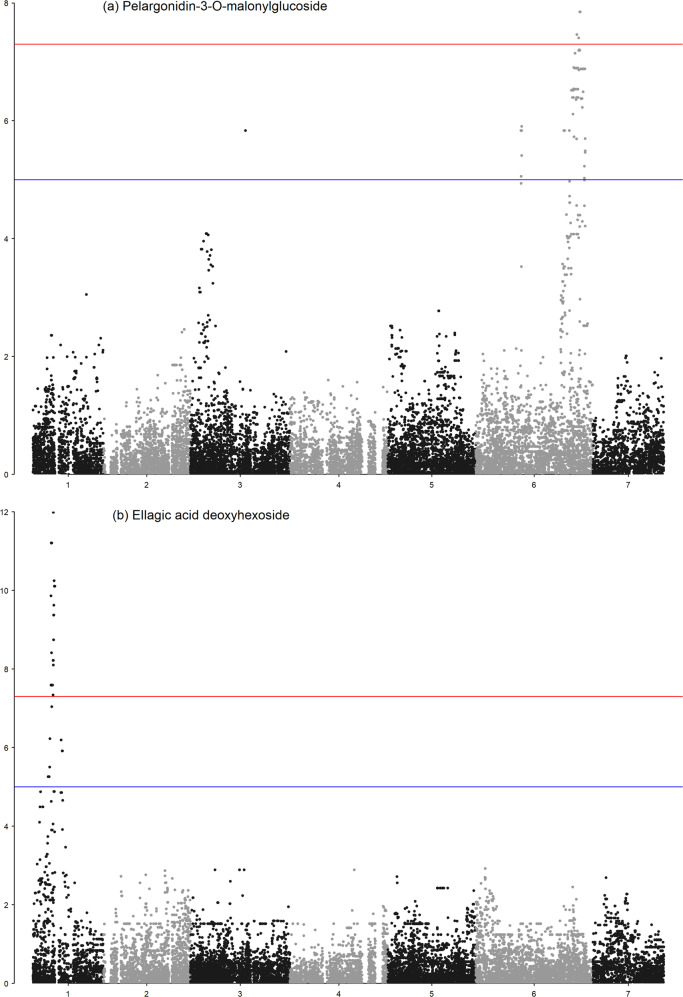
Manhattan plots of markers significantly associated with pelargonidin-3-O-malonylglucoside and ellagic acid deoxyhexoside production in cultivated strawberry. A Manhattan plot constructed against the *F. vesca* v4.0 genome sequence showing marker-trait associations exceeding the −log10(*p*) value significance threshold of 6.5 for the polyphenols **a** pelargonidin-3-*O*-malonylglucoside on *Fvb6*, and **b** ellagic acid deoxyhexoside on *Fvb1* from the combined phenotypic and genotypic data from three cultivated strawberry mapping populations obtained using the MLM method

GWAS were conducted using 24,062 informative markers with their relative positions derived from the *F. vesca* v4.0 reference genome^25^. Of these SNPs, a total of 4,317 were placed reliably on the *F.* × *ananassa* ‘Camarosa’ genome sequence^26^ by Hardigan et al.^27^. The GWAS analyses identified significant marker-trait associations for the polyphenols pelargonidin-3-*O*-malonylglucoside, pelargonidin-3-*O*-acetylglucoside, cinnamoyl glucose, and ellagic acid deoxyhexoside in the combined progenies dataset that exceeded the −log10 (*p*) value significance threshold of 6.5. A total of 163 significant associations were identified with SNP markers for pelargonidin-3-*O*-malonylglucoside (Fig. 2a), 60 were identified for pelargonidin-3-*O*-acetylglucoside (Fig. S1), 33 were identified for ellagic acid deoxyhexoside (Fig. 2b), while a single significant association was determined for cinnamoyl glucose (Fig. S1). The MLM model approach confirmed the associations observed in the basal GLM model in all four instances (Fig. S1). All significant loci identified from the 24,062 informative markers with a position on the *F. vesca* genome were confirmed in the ‘Camarosa’ reference genome (Fig. S2), however, only in the case of pelargonidin-3-*O*-acetylglucoside were the most significant SNPs from the informative GWAS marker set mapped to the ‘Camarosa’ genome. The most significant SNPs at each locus are given in Table 2 along with the physical interval in which all significant markers were located on the *F. vesca* v4.0 reference genome.

**Table 2:** Position of the most significant single-nucleotide polymorphism markers from the iStraw35 array co-segregating with the polyphenols pelargonidin-3-*O*-malonylglucoside, pelargonidin-3-*O*-acetylglucoside, and ellagic acid deoxyhexoside, and the intervals with significant markers on the *F. vesca* v4.0 genome sequence

Polyphenol	Marker	−log_10_(*p*)	Chromsome	Position	Interval of significant markers according to the GWAS
Pelargonidin-3-*O*-malonylglucoside	AX-166507798	11.22	6	34,567,280	28,147,851–36,394,202
	AX-166507810	11.22	6	34,693,440	
	AX-123525466	11.22	6	34,624,092	
Pelargonidin-3-*O*-acetylglucoside	AX-166527347	9.15	6	35,452,042	31,813,892–36,394,202
	AX-166507856	9.15	6	35,391,196	
Ellagic acid deoxyhexoside	AX-166503105	11.91	1	6,707,123	5,933,302–7,230,807

### QTL analysis and mapping of traits under single major gene control

**Fig. 3:**
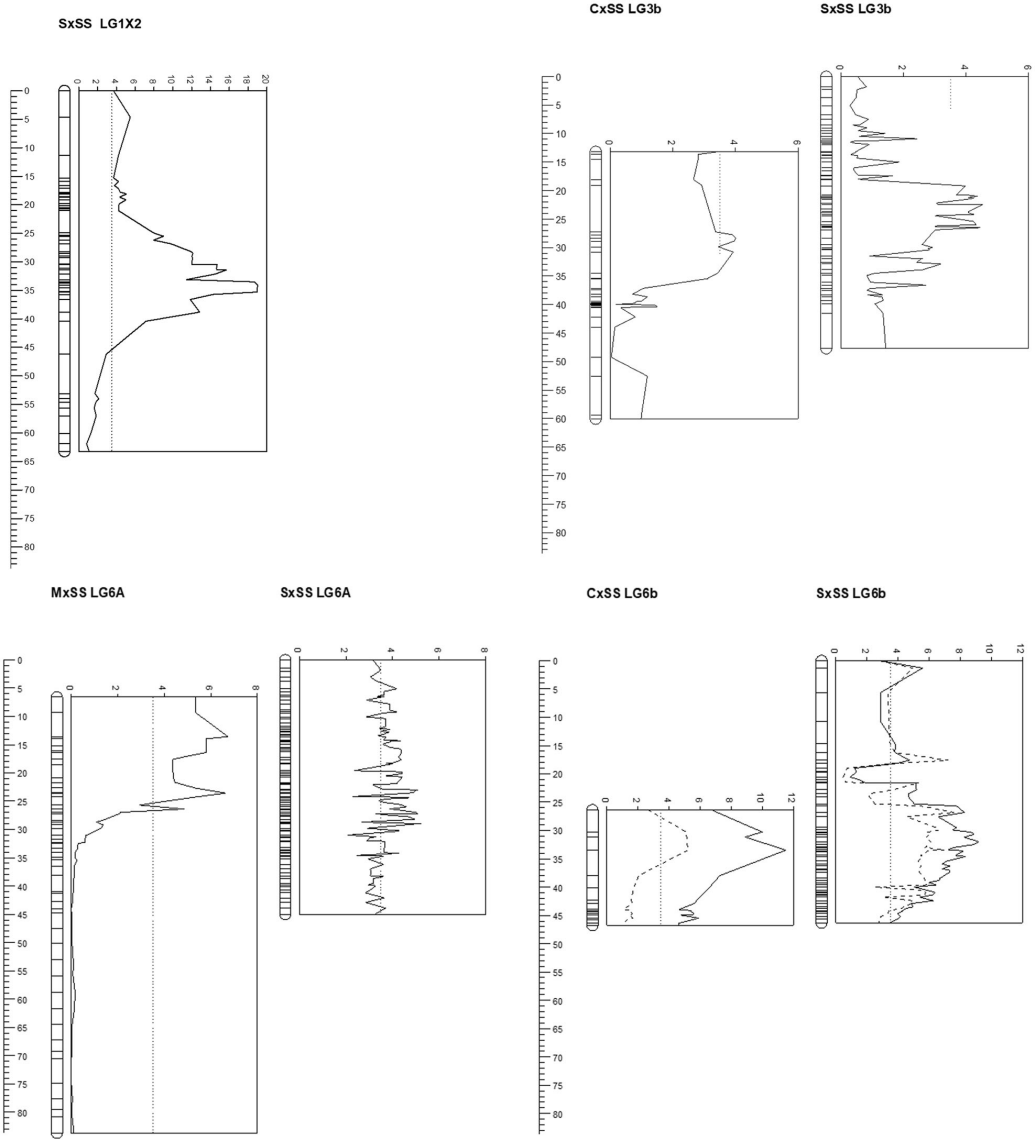
QTL plots for ellagic acid deoxyhexoside, cinnamoyl glucoside and pelargonidin-3-O-malonylglucoside identified in cultivated strawberry. Plots of significant QTL (LOD threshold >3.5) on **a** LG1X of the S×SS mapping population (‘Camarosa’ *Fvb1-3*) for ellagic acid deoxyhexoside; **b** LG3b of the C×SS and S×SS mapping populations (‘Camarosa’ *Fvb3-2*) for cinnamoyl glucoside; **c** LG6A of the M×SS and S×SS mapping populations (‘Camarosa’ *Fvb6-1*) for cinnamoyl glucoside; **d** LG6b of the C×SS and S×SS mapping populations (‘Camarosa’ *Fvb6-2*) for pelargonidin-3-*O*-malonylglucoside (unbroken line) and pelargonidin-3-*O*-acetylglucoside (hashed line). LOD scores are plotted along the *x*-axes (dotted line indicates the 3.5 LOD threshold), while genetic distances (cM) are plotted along the *y*-axes. Positions of each homologous linkage group relative to each other in the different maps follow the physical positions of the linkage groups on the ‘Camarosa’ genome sequence

Significant QTL were identified corresponding to the genomic intervals containing GWAS associations for pelargonidin-3-*O*-malonylglucoside, pelargonidin-3-*O*-acetylglucoside, cinnamoyl glucose, and ellagic acid deoxyhexoside (Fig. 3). A significant QTL was identified on LG1X2 of the ‘Saga’ × ‘Senga Sengana’ (S×SS) mapping population for ellagic acid deoxyhexoside; significant QTL were identified for cinnamoyl glucose on linkage groups LG3b in the ‘Carisma’ × ‘Senga Sengana’ (C×SS) and S×SS mapping populations and LG6A in the ‘Marlate’ × ‘Senga Sengana’ (M×SS) and S×SS populations, and significant QTL were identified for pelargonidin-3-*O*-malonylglucoside and pelargonidin-3-*O*-acetylglucoside on LG6b of the C×SS and S×SS populations (Table 3). Axiom marker data presented by Hardigan et al.^27^ enabled linkage groups on the three mapping populations to be assigned to chromosome sequences on the ‘Camarosa’ cultivated strawberry reference genome sequence of Edger et al.^26^. Between the three mapping populations, 51 markers from linkage group LG1X2 were anchored to ‘Camarosa’ chromosome Fvb1-3, 59 markers were anchored to chromosome Fvb3-2, 24 markers were anchored to Fvb6-1, and 22 markers were anchored to Fvb6-2. No ambiguities were observed within linkage groups or between homologous groups between mapping populations relating to the chromosomes to which they were anchored on the ‘Camarosa’ genome sequence.

**Table 3:** QTL identified in the three mapping progeny detailing the mapping population in which they were identified, the linkage group, the marker(s) most significantly associated with the variance observed, the LOD, the percentage variance explained and the genetic and physical positions of the peak LOD of each QTL on the ‘Camarosa’ genome sequence where an association was found

Trait	Mapping population	Linkage group	Most significant marker(s)	LOD	Observed variance explained (%)	Genetic distance (cM)	‘Camarosa’ chrom	Physical postion (Mbp)
Ellagic acid deoxyhexoside	‘Saga’ × ‘Senga Sengana’	LG1X2	AX-123357156	19.01	86.3	34.1	Fvb1-3	7.16
Cinnamoyl glucoside	‘Carisma’ × ‘Senga Sengana'	LG3b	AX-123361118	4.01	32.5	16.7	Fvb3-2	3.82
	‘Saga’ × ‘Senga Sengana’	LG3b	AX-89787536	4.36	36.6	21	Fvb3-2	7.59
	‘Marlate’ × ‘Senga Sengana'	LG6A	AX-123615126, AX-166524901	6.63	51.7	17.1	Fvb6-1	17.29
	‘Saga’ × ‘Senga Sengana’	LG6A	AX-166524984	5.1	41.2	22.6	Fvb6-1	15.58
Pelargonidin-3-*O*-malonylglucoside	‘Carisma’ × ‘Senga Sengana'	LG6b	AX-166519413, AX-166507849, AX-166515515, AX-166516086	11.51	67.6	7.1	Fvb6-2	30.1–31.7
	‘Saga’ × ‘Senga Sengana’	LG6b	AX-123362709, AX-166507798	9.19	61.8	32	Fvb6-2	31.23–31.27
Pelargonidin-3-*O*-acetylglucoside	‘Carisma’ × ‘Senga Sengana'	LG6b	AX-166519413, AX-166507849, AX-166515515, AX-166516086	5.21	40.4	7.1	Fvb6-2	30.1–31.7
	‘Saga’ × ‘Senga Sengana’	LG6b	AX-166505280	7.63	60.8	26.812	Fvb6-2	N/A^a^

^a^No significant BLAST hit on chromosome Fvb6-2

**Fig. 4:**
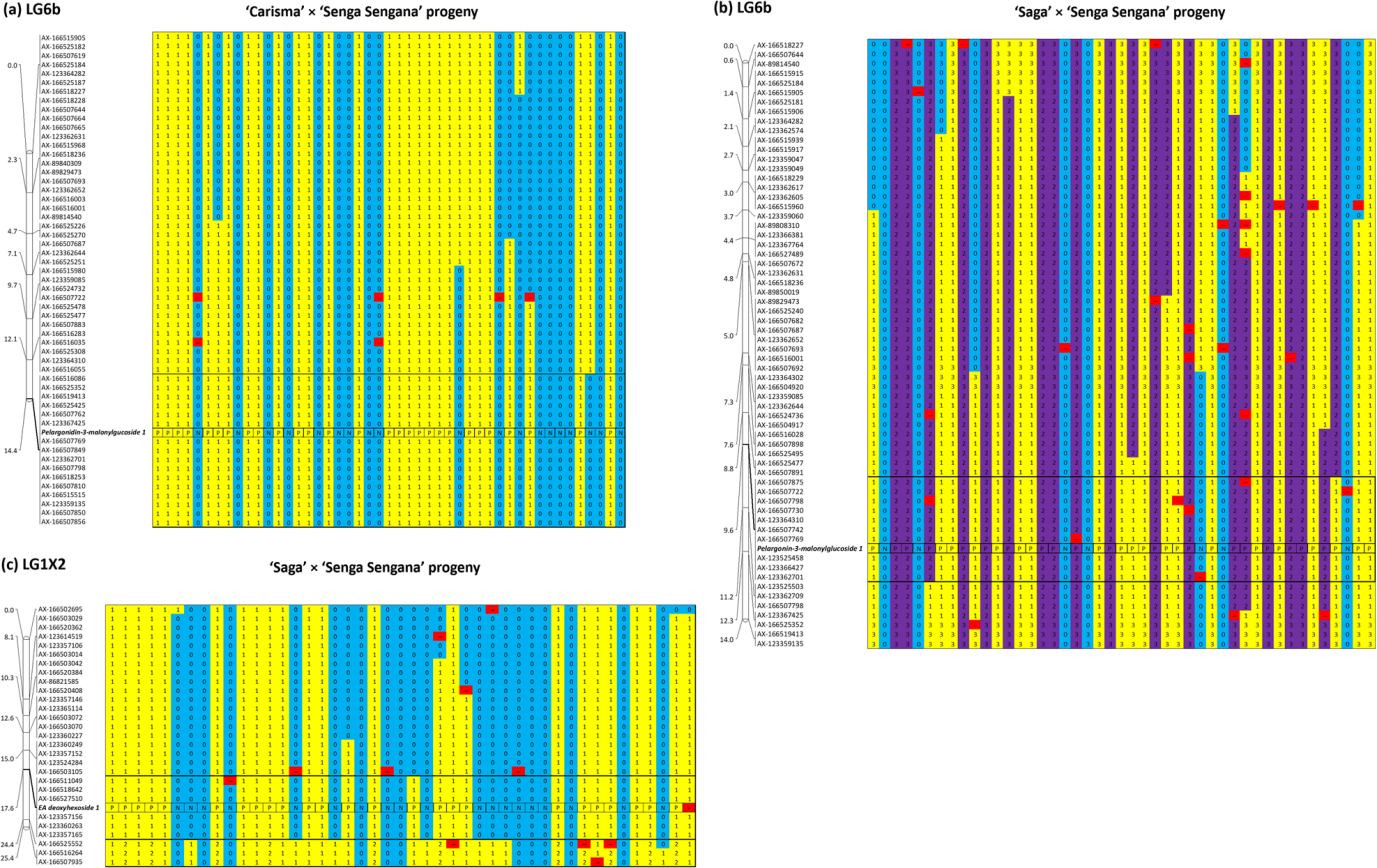
Plot showing the genetic positions and marker genotypes of major genes controlling pelargonidin-3-O-malonylglucoside and ellagic acid deoxyhexoside in cultivated strawberry. Genetic data for LG6b of the **a** C×SS and **b** S×SS linkage maps detailing the genetic position of the locus controlling the biosynthesis of pelargonidin-3-*O*-malonylglucoside and **c** LG1X2 of the S×SS mapping population detailing the genetic position of the locus controlling ellagic acid deoxyhexoside when the seedling phenotypes were scored qualitatively as ‘producers (P) or ‘non-producers (N). Genotypes 0 and 2 denote homozygotes (AA or BB), 1 denotes heterozygotes (AB) and 3 denotes dominant markers where the allele is present (AA, A_, BB or B_)

Following GWAS and QTL analysis, the concentrations of ellagic acid deoxyhexoside observed in ripe fruit of the progeny of the S×SS population, and pelargonidin-3-malonylglucoside observed in ripe fruit of the progeny of the C×SS and S×SS mapping populations were scored as a qualitative presence/absence phenotype and linkage mapping confirmed discrete genetic positions of the traits indicating that they were under the control of a single major gene in these populations. Loci controlling ellagic acid deoxyhexoside and pelargonidin-3-*O*-malonylglucoside biosynthesis co-segregated with markers mapped to genomic intervals on chromosome *Fvb1-3* of the ‘Camarosa’ genome sequence between 6,921,203 bp and 7,554,779 bp (an interval of 633,576 bp) and chromosome *Fvb6-2* of the ‘Camarosa’ genome sequence between 31,229,150 bp and 32,997,567 bp (an interval of 1,768,417 bp), respectively (Fig. 4), according to the physical positions of markers with which they co-segregated. These regions corresponded to a genomic interval between 6,707,123 bp and 8,633,460 bp (1,781,033 bp) on *Fvb1* and an interval between 34,188,443 bp and 35,448,208 bp (1,259,765 bp) on *Fvb6* of the of the *F. vesca* v4.0 genome sequence, respectively.

**Fig. 5:**
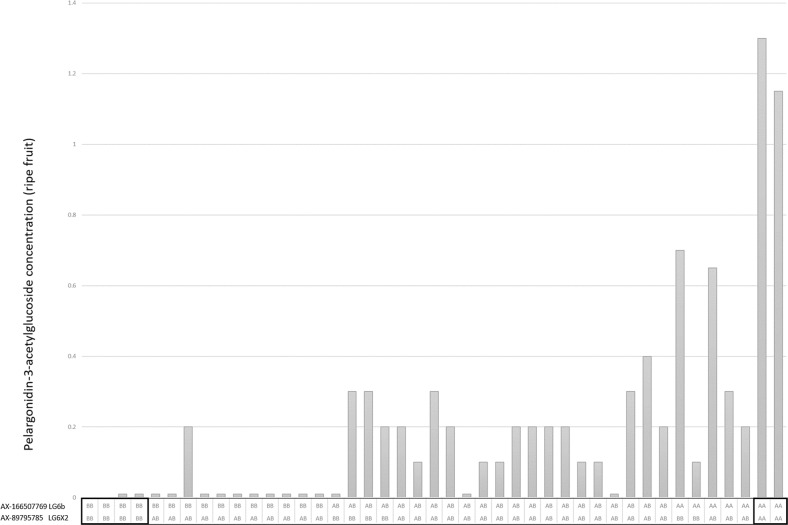
Plot showing the relationship between pelargonidin-3-*O*-acetylglucoside concentrations and the genotype classes at two unlinked loci on LG6X2 and LG6b in the S×SS mapping population. The *x* axis shows the genotype classes for markers AX-166507769 and AX-89795785 and the *y* axis shows the pelargonidin-3-*O*-acetylglucoside concentrations in mg 100 g^−1^ FW. Double homozygous genotype classes in the progeny of the two markers on LG6X2 and LG6b produced the highest and lowest concentrations of pelargonidin-3-*O*-acetylglucoside in the ripe fruit of the progeny

The QTL for pelargonidin-3-*O*-malonylglucoside and pelargonidin-3-*O*-acetylglucoside were identified in the same region of linkage group LG6b in both the C×SS and S×SS linkage maps (Fig. 3). However, when phenotypic data for pelargonidin-3-*O*-acetylglucoside were scored qualitatively, the segregation data did not fit the hypothesis of a single major gene controlling the biosynthesis of this compound in either population. A second significant QTL for pelargonidin-3-*O*-acetylglucoside biosynthesis was identified on LG6X2 in the S×SS mapping population. While marker genotypes were not completely predictive of phenotype, due to the genetic distance between mapped markers and the genetic loci controlling the trait variation, the combination of homozygous genotypes in this progeny of the most significantly associated markers on both LG6X2 and LG6b produced the highest and lowest concentrations of pelargonidin-3-*O*-acetylglucoside in the ripe fruit of the progeny (Fig. 5). The LG6X2 QTL was not recovered in the C×SS progeny.

**Fig. 6:**
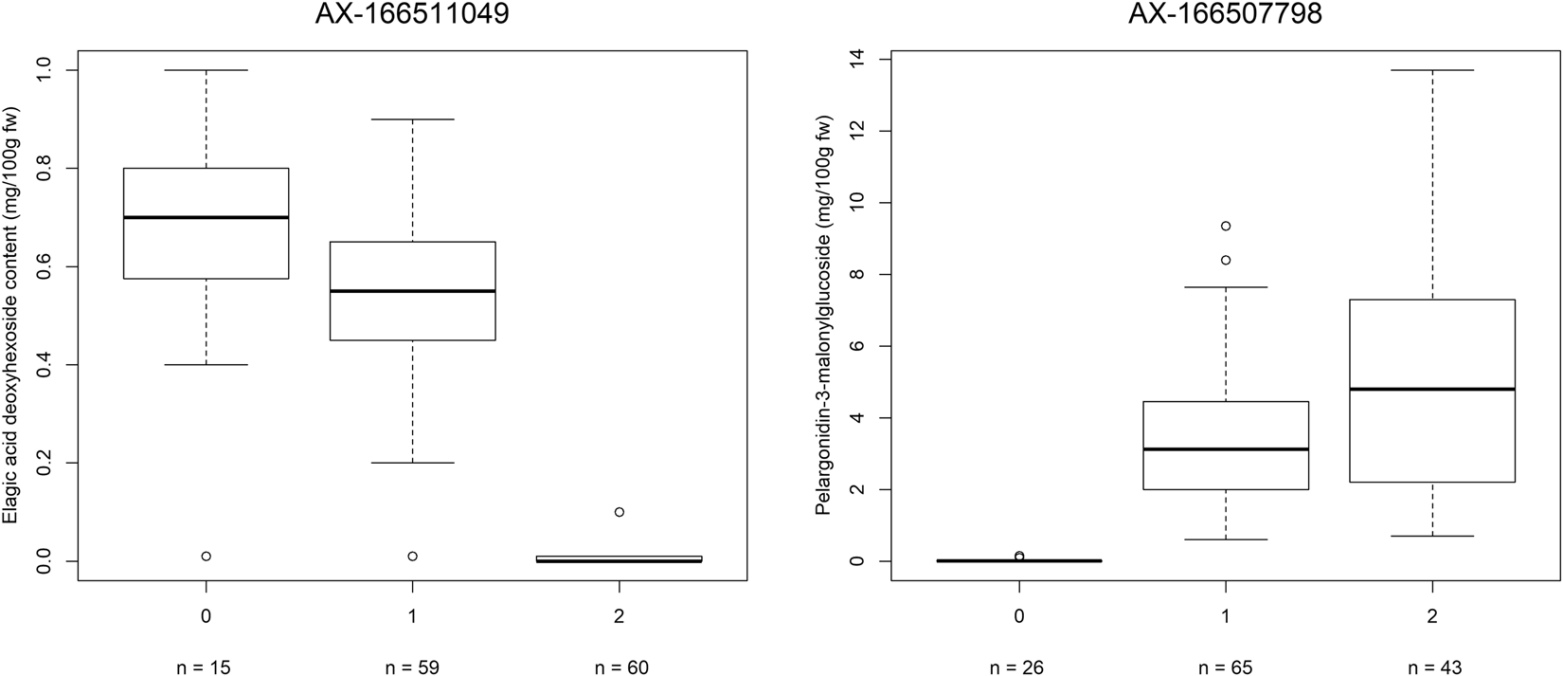
Allele-effect box plots of ellagic acid deoxyhexoside and pelargonidin-3-O-malonylglucoside concentrations for two Axiom markers. SNP markers **a** AX-166511049 and **b** AX-166507798 co-segregating with quantitative phenotypic trait scores for ellagic acid deoxyhexoside and pelargonidin-3-*O*-malonylglucoside, respectively. Marker classes are as follows: 0=AA genotype, 1=AB, and 2=BB genotype according to the data of Verma et al.^51^

Allele-effect box plots of SNP markers co-segregating in individual populations with qualitative phenotypic trait scores for ellagic acid deoxyhexoside and pelargonidin-3-*O*-malonylglucoside (AX-166511049 and AX-166507798, respectively) are shown in Fig. 6. The allele effects for SNPs co-segregating with pelargonidin-3-*O*-malonylglucoside and ellagic acid deoxyhexoside were predictive of polyphenol concentrations across all three mapping populations.

### Candidate gene identification

Initially, a search for candidate genes was performed within the major gene intervals identified in the *F. vesca* v4.0 genome sequence where a total of 561 gene predictions were identified in the 3 Mb genomic interval (6–9 Mb) on *Fvb1* containing the locus controlling ellagic acid deoxyhexoside biosynthesis, and 408 gene predictions were identified in the 2 Mb genomic interval (34–36 Mb) on *Fvb6* containing the locus controlling pelargonidin-3-*O*-malonylglucoside biosynthesis. Of those on *Fvb1*, a single gene was identified as a potential candidate for ellagic acid deoxyhexoside biosynthesis, while seven genes were identified as candidates for pelargonidin-3-*O*-malonylglucoside biosynthesis on *Fvb6* (Table 4).

**Table 4:** Candidate genes proposed for ellagic acid deoxyhexoside and pelargonidin-3-*O*-malonylglucoside biosynthesis including the chromosome on which they were predicted, their genomic positions, NCBI accession numbers and NCBI annotation description

Gene prediction	Chrom.	Start	End	Strand	Transcript ID	NCBI nr	Description
* Ellagic acid deoxyhexoside*							
FvH4_1g12660.t1	Fvb1	6,961,036	6,962,472	−	FvH4_1g12660.t1_v4.0.a2	XP_004287868.1	putative UDP-rhamnose:rhamnosyltransferase 1 [*Fragaria vesca* subsp. *vesca*]
* Pelargonidin-3-O-malonylglucoside*							
FvH4_6g46740.t1	Fvb6	35,572,100	35,574,163	+	FvH4_6g46740.t1_v4.0.a2	XP_004306064.1	phenolic glucoside malonyltransferase 1-like [*Fragaria vesca* subsp. *vesca*]
FvH4_6g46741.t1	Fvb6	35,575,340	35,576,509	+	FvH4_6g46741.t1_v4.0.a2	XP_011468648.1	malonyl-CoA:anthocyanidin 5-O-glucoside-6″-O-malonyltransferase-like [*Fragaria vesca* subsp. *vesca*]
FvH4_6g46742.t1	Fvb6	35,577,651	35,579,147	+	FvH4_6g46742.t1_v4.0.a2	XP_004304224.1	phenolic glucoside malonyltransferase 1-like [*Fragaria vesca* subsp. *vesca*]
FvH4_6g46743.t1	Fvb6	35,580,693	35,585,111	+	FvH4_6g46743.t1_v4.0.a2	XP_004304225.1	phenolic glucoside malonyltransferase 1-like [*Fragaria vesca* subsp. *vesca*]
FvH4_6g46750.t1	Fvb6	35,585,454	35,586,797	+	FvH4_6g46750.t1_v4.0.a2	XP_004306067.1	phenolic glucoside malonyltransferase 1-like [*Fragaria vesca* subsp. *vesca*]
FvH4_6g46770.t1	Fvb6	35,594,333	35,596,185	+	FvH4_6g46770.t1_v4.0.a2	XP_011467880.1	phenolic glucoside malonyltransferase 1-like [*Fragaria vesca* subsp. *vesca*]
FvH4_6g46780.t1	Fvb6	35,603,196	35,605,063	+	FvH4_6g46780.t1_v4.0.a2	XP_011467881.1	phenolic glucoside malonyltransferase 1-like [*Fragaria vesca* subsp. *vesca*]

Gene FvH4_1g12660, located in the *Fvb1* interval and annotated as a putative UDP-rhamnose: rhamnosyltransferase 1 was identified as the most likely candidate for the gene controlling ellagic acid deoxyhexoside biosynthesis. Transcript abundance data were not available for the cultivated strawberry species *F.×ananassa* for the candidate genes identified, however, data for the related diploid species *F. vesca*, housed on the *F. vesca* eFP browser^16^ (bioinformatics.towson.edu/strawberry) showed that the gene was differentially expressed during fruit development in the *F. vesca* cultivars ‘Ruegen’ and ‘Yellow Wonder’ with transcript levels observed in ‘Yellow Wonder’ ripe fruit higher than in ‘Ruegen’ (Table 5).

**Table 5:** Relative transcript abundance of the eight candidate genes identified in this investigation in the *F. vesca* cultivars ‘Ruegen’ (red-fruited) and ‘Yellow Wonder’ (white-fruited) during fruit development

Gene	RPKM		RPKM		RPKM		RPKM	
	Ruegen, 15d	Std dev.	Ruegen, Turning	Std dev.	Yellow Wonder, 15d	Std dev.	Yellow Wonder, Turning	Std dev.
*UDP-glucose glucosyltransferase (FaGT1)*								
FvH4_7g33840	1.51	0.88	106.8	46.24	1.3	0.26	0.27	0.03
*Pelargonidin-3-O-malonylglucoside*								
FvH4_6g46740.t1	0.11	0.05	0.04	0.01	0.1	0.03	0.02	0.02
FvH4_6g46741.t1	5.27	1.52	4.82	0.43	4.07	0.63	4.07	1.71
FvH4_6g46742.t1	0	0	0.02	0.02	0	0	0	0
FvH4_6g46743.t1	9.81	0.24	6	0.41	8.83	0.53	4.07	0.96
FvH4_6g46750.t1	2.98	0.64	4.29	0.66	2.32	0.01	3.17	1.53
FvH4_6g46770.t1	0.08	0.03	0.01	0.01	0.29	0.08	0.01	0.01
FvH4_6g46780.t1	5.99	1.46	6.25	0.17	3.72	0.47	3.13	1.88
Ellagic acid deoxyhexoside								
FvH4_1g12660	23.05	8.56	39.61	8.45	18.39	0.42	63.34	2.3

Data taken from the eFP browser (bioinformatics.towson.edu/strawberry) of Hawkins et al.^10^

Within the *Fvb6* interval, seven candidate genes (FvH4_6g46740.t1, FvH4_6g46741.t1, FvH4_6g46742.t1, FvH4_6g46743.t1, FvH4_6g46750.t1, FvH4_6g46770.t1, and FvH4_6g46780.t1) annotated with malonyltransferase activity were identified as likely candidates for the gene controlling pelargonidin-3-*O*-malonylglucoside biosynthesis. Transcript abundance data located on the strawberry eFP browser (bioinformatics.towson.edu/strawberry)^16^ from ripening fruit tissue of *F. vesca* cultivars ‘Ruegen’ and ‘Yellow Wonder’ was scrutinized for the seven candidate genes identified, and compared with transcript abundance data in the same tissues for gene FvH4_7g33840, the glycosyltransferase shown previously to be responsible for the production of pelargonidin-3-*O*-glucoside in *F.* × *ananassa* and *F. vesca*^28^. Different transcript abundance patterns were observed for each of the candidate genes (Table 5). Genes FvH4_6g46741.t1, FvH4_6g46743.t1, FvH4_6g46750.t1, and FvH4_6g46780.t1 showed relatively high transcript levels during fruit ripening. Gene FvH4_6g46750.t1 was upregulated during ripening in both ‘Ruegen’ (red-fruited) and ‘Yellow Wonder’ (white-fruited), but more strongly upregulated in ‘Ruegen’, while gene FvH4_6g46780.t1 was not significantly upregulated in either cultivar, but showed significantly higher transcript levels in ‘Ruegen’ than in ‘Yellow Wonder’. Between the same tissues, gene FvH4_7g33840 (FvGT1) was significantly upregulated during fruit development, but only in the red-fruited ‘Ruegen’ cultivar (Table 5).

Following identification and annotation of candidate genes in the *F. vesca* v4.0 genome sequence, gene predictions within the major gene intervals in the ‘Camarosa’ genome sequence were annotated, and a total of 361 gene predictions were identified in the 2.5 Mb genomic interval (6.5–9 Mb) on *Fvb1-3* containing the locus controlling ellagic acid deoxyhexoside biosynthesis, and 384 gene predictions were identified in the 2.5 Mb genomic interval (31–33.5 Mb) on *Fvb6-2* containing the locus controlling pelargonidin-3-*O*-malonylglucoside biosynthesis.

A search was then made for homologous genes between ‘Camarosa’ and *F. vesca* at the major gene loci for ellagic acid deoxyhexoside and pelargonidin-3-*O*-malonylglucoside biosynthesis identified using the Tripal synteny viewer implemented on the Genome Database for Rosaceae^29^. The syntenic block fafvB1334 identified between chromosomes *Fvb1* of the *F. vesca* v4.0 genome sequence and *Fvb1-3* of the ‘Camarosa’ genome sequence containing the gene controlling ellagic acid deoxyhexoside biosynthesis spanned the region 6,445,166–16,286,418 (10.8 Mb) in *F. vesca* and 5,367,671–16,828,623 (11.5 Mb) in ‘Camarosa’ and displayed a high degree of overall synteny (bit score = 39,011 e-value = 0). The syntenic block fafvB0858 identified between chromosomes *Fvb6* of the *F. vesca* v4.0 genome sequence and *Fvb6-2* of the ‘Camarosa’ genome sequence containing the gene controlling pelargonidin-3-*O*-malonylglucoside biosynthesis spanned the region 30,223,352–36,410,328 (6.2 Mb) in *F. vesca* and 31,545,552–36,081,520 (5.5 Mb) in ‘Camarosa’ and also displayed a high degree of overall synteny (bit score = 24,204 e-value = 0). Within the *Fvb1-3* syntenic block, there were 1076 ‘Camarosa’ and 1378 *F. vesca* genes, of which 787 were identified as homologous, while in the *Fvb6-2* syntenic block, there were 681 ‘Camarosa’ and 949 *F. vesca* genes, of which 492 were identified as homologous. Homologous gene sequences were identified for two of the candidate genes on *Fvb6*; FvH4_6g46750.1 most closely aligned to maker-Fvb6-2-snap-gene-312.67-mRNA-1 (e-value = 1e−128) and FvH4_6g46740.1 most closely aligned to maker-Fvb6-2-snap-gene-312.68-mRNA-1 (e-value = 0). No homologous gene was identified for the candidate gene on *Fvb1*, FvH4_1g12660.t1_v4.0.a2.

## Discussion

Polyphenol compounds are emerging as potent types of phytochemicals with pleiotropic effects on human health imparted through a dynamic interaction with the gut microbiome. Ingested polyphenols modulate microbiota community composition while microbiota enzymatically transform polyphenols into bioavailable compounds with a range of activities including anti-inflammatory and neuroprotective effects^5^. It is to be expected then, that fruit with increased polyphenol compounds might come into focus of breeding efforts for crops worldwide, including strawberries and other Rosaceous crops. Hence it is timely and of importance to improve our understanding of the genetics basis of polyphenol biosynthesis and accumulation in crops used for human consumption.

The genetics of specific polyphenol compound biosynthesis were investigated in cultivated strawberry here for the first time using three mapping populations raised from parental genotypes previously shown to differ in the polyphenol content of their berries^10^. The concentrations of phenolic compounds observed in the parental lines and progenies were in the range previously reported in strawberries^10–12^ and significant QTL were identified for four of these polyphenols in the mapping populations studied; pelargonidin-3-*O*-malonylglucoside, pelargonidin-3-*O*-acetylglucoside, cinnamoyl glucose, and ellagic acid deoxyhexoside. Moreover, the concentrations of two of the compounds in the fruits of the mapping populations: pelargonidin-3-*O*-malonylglucoside and ellagic acid deoxyhexoside, were mapped as qualitative traits and were shown to be controlled by a single major gene.

### Genetic control of ellagic acid deoxyhexoside production

Deoxyhexoses are a class of six-carbon monosaccharides that have had one or more of their hydroxyl groups replaced with hydrogen atoms and include rhamnose, arabinose, quinovose and fucose. Aaby et al.^9^ reported the presence of ellagic acid deoxyhexoside in the ripe fruits of the cultivated strawberry, with subsequent studies also reporting the presence of the compound in strawberry fruit^10–12,30^. Far less is known about the biosynthesis of ellagitannins than about the biosynthesis of phenylpropanoids, flavonoids, and anthocyanins. Gallic acid is the basic precursor of ellagitannin biosynthesis, and esterification of gallic acid and uridine-5′-diphosphate glucose (UDP-glucose) by 1-*O*-acylglucose glucosyltransferases leads to the formation of *β*-glucogallin^31^, which is converted to 1,2,3,4,6-pentagalloylglucose by 1-*O*-acylglucose dependent acyltransferases^32^. From here it is suggested that 3,4,5,3′,4′,5′-hexahydroxydiphenoyl moieties are produced through oxidation^33^, and that ellagic acid is then formed by hydrolysis^34^. A recent study in wild and cultivated *Fragaria* species characterized five 1-*O*-acylglucose glucosyltransferases^35^, with one gene FaGT2, physically located at 4,152,828 bp on Fvb2 of the *F. vesca* v4.0 genome sequence, shown to be responsible for *β*-glucogallin biosynthesis.

To date, other genes producing proteins that catalyze reactions within the ellagitannin biosynthesis pathway have not been characterized, nor have genes responsible for the formation of ellagic acid deoxyhexoside. However, Urrutia et al.^17^ reported a major QTL for ellagic acid biosynthesis in diploid *Fragaria* in the NIL interval Fb1.26-61, which spanned the physical interval between 3,315,998 and 20,747,404 bp on the diploid *Fragaria* genome, and was therefore not the FaGT2 locus reported by Schulenburg et al.^35^. The 633 kb region mapped in this investigation is within the physical interval of the QTL identified by Urrutia et al.^17^. However, it is unlikely that the two loci are orthologous because there was no correlation between the concentrations of ellagic acid and ellagic acid deoxyhexoside in fruits of the S×SS mapping population. Moreover, the lack of an identifiable QTL for ellagic acid production on LG1X2 in this investigation suggests that they are under independent genetic control. Since ellagic acid and ellagic acid deoxyhexoside concentrations were not correlated, we hypothesized that the gene underlying the locus identified on LG1X2 in this investigation catalyzed the glycosylation of ellagic acid using a deoxyhexose sugar substrate, leading to the formation of ellagic acid deoxyhexoside.

Within the physical interval characterized in this investigation in *F. vesca*, a single candidate gene with a putative role in ellagic acid deoxyhexoside biosynthesis, FvH4_1g12660, was identified. Gene FvH4_1g12660 was annotated as a putative UDP-rhamnose:rhamnosyltransferase 1, which has previously been shown to be involved in flavonoid modification in *Lobelia erinus*^36^. Rhamnose is a deoxyhexose sugar that has been shown to be present in the ripe fruits of the cultivated strawberry^37^. Ellagic acid rhamnosides have been identified in the stem bark of *Syzygium guineese*^38^ and more recently in the fruits of *Rubus ulmifolius*^39^, a close relative of the genus *Fragaria*. It is therefore plausible that the ellagic acid deoxyhexoside produced in cultivated strawberry is ellagic acid rhamnoside and that its biosynthesis is under the control of the candidate gene FvH4_1g12660. While caution should be exercised when comparing data between related species, the transcript abundance profile of FvH4_1g12660 in *F. vesca* showed that it was upregulated during fruit development, and that transcript levels in the white-fruited cultivar ‘Yellow Wonder’ were higher than those observed in the red-fruited cultivar ‘Ruegen’ (Table 5). This observation was consistent with the findings of Roy et al.^40^ who reported a greater accumulation of ellagitannins in white-fruited over the red-fruited *F. vesca* cultivars. Taken together, the physical location of the FvH4_1g12660 gene, and previously reported transcript profiles for *F. vesca* suggests gene FvH4_1g12660 as a candidate for ellagic acid deoxyhexoside biosynthesis in cultivated strawberry on LG1X2, particularly as no other genes in the mapping interval were potentially involved in catalyzing glycosylation reactions. However, while full-length homologs of the FvH4_1g12660 gene were identified within the physical interval on *Fvb1* homeologues *Fvb1-1* and *Fvb1-2* in the ‘Camarosa’ genome, the *Fvb1-3* homolog of the gene was absent from the ‘Camarosa’ assembly. No reports have been published to date as to whether ‘Camarosa’ is an ellagic acid deoxyhexoside producer; if it is a non-producer, it is possible that the complete deletion of the gene from the *Fvb1-3* chromosome is responsible for the lack of ellagic acid deoxyhexoside production in some cultivated strawberry accessions. Further analyses need to be performed to functionally characterize the gene and its expression in cultivated strawberry, and demonstrate if it has a role in controlling ellagic acid deoxyhexoside biosynthesis, or if another candidate gene in this region is the causal genetic agent.

### Genetic control of pelargonidin-3-*O*-malonylglucoside formation

Anthocyanins are the class of pigments that give ripe strawberry fruits their red color and their concentrations significantly vary between cultivars. The total anthocyanin content in strawberry fruits is predominantly composed of pelargonidin-3-*O*-glucoside, pelargonidin-3-*O*-malonylglucoside, cyanidin-3-*O*-glucoside and pelargonidin-3-*O*-rutinoside, and the balance between these anthocyanins affect the color of the ripe berries^23,41^. Pelargonidin-3-*O*-malonylglucoside was first identified in strawberry fruits by Tamura et al.^42^, who noted its presence in the Japanese cultivars ‘Nyoho’ and ‘Reiko’, but did not detect it in ‘Ai-berry’ or ‘Toyonoka’. Later, Yoshida et al.^23^ studied the levels of pelargonidin-3-*O*-malonylglucoside in relation to fruit color in 20 cultivars of mainly Japanese origin and reported that nine of the cultivars studied were non-producers, while the remaining eleven were producers. In more recent studies, pelargonidin-3-*O*-malonylglucoside was determined to be the second most abundant anthocyanin in ripe red fruits of 27^10^ and 90^12^ strawberry cultivars, with concentrations ranging from 0.0 to 20.8 mg 100 g^−1^ of FW. The results of these studies, demonstrating the presence or absence of the production of pelargonidin-3-*O*-malonylglucoside between strawberry cultivars, suggests that a mutation in a single major gene is responsible for the absence of the compound in some cultivars. However, to date, the inheritance and genetic control of pelargonidin-3-*O*-malonylglucoside has not been studied in the cultivated strawberry.

Here, a major QTL for the presence/absence of pelargonidin-3-*O*-malonylglucoside was observed, and a qualitative interpretation of segregation data from two mapping populations (C×SS and S×SS), demonstrated the presence of a mutation in a single major gene locus determining the lack of biosynthesis of the compound in a 1,768,417 bp interval on *Fvb6-2* of the *F. vesca* genome between 31,229,150 and 32,997,567 bp. Lerceteau-Köhler et al.^18^ identified a QTL for total anthocyanin content on LGVIb of the ‘Capitola’ × ‘CF1116’ mapping population, and identified the microsatellite marker EMFv010^43^ as the transferrable genetic marker most closely associated to the trait. While the precise location of the QTL was not reported in that study, the physical position of EMFv010 on *Fvb6* of the *F. vesca* v4.0 genome sequence is 31,622,577 bp, which is within the genetic interval defined in this investigation. More recently, Urrutia et al.^17^ studied the inheritance of numerous polyphenol compounds in an interspecific diploid near isogenic line population, and mapped a minor QTL for pelargonidin-3-*O*-malonylglucoside explaining 10% of the observed phenotypic variation to a 6.4 Mb genomic interval between 32,907,471 and 39,317,498 bp on *Fvb6* of the *F. vesca* v4.0 genome sequence, which encompasses the interval defined in this investigation. Thus it is highly likely that an orthologous locus controls pelargonidin-3-*O*-malonylglucoside production in diploid and octoploid *Fragaria*.

Pelargonidin-3-*O*-malonylglucoside in cultivated strawberry is synthesized via pelargonidin, which is converted to pelagonidin-3-*O*-glucoside by the activity of anthocyanidin glucosyltransferase FaGT1. Subsequently, malonylation is achieved by a previously uncharacterized malonyltransferase to give pelargonidin-3-*O*-malonylglucoside^44^. Our investigation revealed that the production of pelargonidin-3-*O*-malonylglucoside in cultivated strawberry is the result of the action of a mutation in a single major gene which determines the qualitative presence or absence of the compound, while the concentration of pelargonidin-3-*O*-glucoside remains relatively unchanged. It has been previously reported that silencing of the anthocyanidin glucosyltransferase GT1 in *F.* × *ananassa* leads to a reduction in the levels of both pelargonidin-3-*O*-malonylglucoside and pelargonidin-3-*O*-glucoside in strawberry fruits, and that the transcript levels of FaGT1 increase as ripening progresses, with highest transcript abundance in ripe red berries^28^. The FaGT1 locus is located on *Fvb7* of the *Fragaria* genome, and is thus not the locus controlling pelargonidin-3-*O*-malonylglucoside biosynthesis. Given that the locus on *Fvb1* identified in this investigation does not affect pelargonidin-3-*O*-glucoside biosynthesis, we postulated that the locus we identified on LG6b of the S×SS mapping population was likely to be the malonyltransferase gene catalyzing this final step of the pathway as described above.

The enzyme malonyl-CoA:anthocyanin 5-*O*-glucoside-6-*O*-malonyltransferase was first shown to catalyze the malonylation (or aliphatic acylation) of anthocyanins in plants by Suzuki et al.^45^ in scarlet sage (*Salvia splendens*) and was more recently characterized in *Arabidopsis* (At5MAT^46^), where it was shown to be the gene responsible for synthesizing malonyl-modified anthocyanins. Dissection of the genetic interval on LG6b revealed a total of seven candidate genes with a putative role in polyphenol production. Each of these candidate genes was annotated as having malonyltransferase activity, with high homology to a predicted malonyl-CoA:anthocyanin 5-*O*-glucoside-6-*O*-malonyltransferase. The data of Hawkins et al.^16^ from previous expression analyses in cultivars of the diploid strawberry *F. vesca* showed that three of the seven candidate genes for pelargonidin-3-*O*-malonylglucoside biosynthesis identified here at the LG6b locus were highly expressed in ripening fruit tissues.

An evaluation of the physical region controlling pelargonidin-3-*O*-malonylglucoside biosynthesis on chromosome *Fvb6-2* on the ‘Camarosa’ genome revealed two predicted genes with orthology to five of those predicted to have malonyltransferase activity from the *F. vesca* genome sequence. These three genes were the most likely candidates from the ‘Camarosa’ physical interval, and thus, we propose one of these three genes as the malonyltransferase that catalyzes the formation of pelargonidin-3-*O*-malonylglucoside from pelargonidin-3-*O*-glucoside. Due to the octoploid nature of the ‘Camarosa’ genome, further genetic characterization of this gene region, and functional characterization of these candidates in producing and non-producing *F.×ananassa* germplasm is required to demonstrate thoroughly their role in synthesizing malonyl-modified anthocyanins in the ripe fruit of cultivated strawberry.

It is also likely that one of the identified candidate genes at the *Fvb6-2* locus was responsible for pelargonidin-3-*O*-acetylglucoside production in the C×SS and S×SS mapping populations, in tandem with a second genetic factor mapped to LG6X2 in the S×SS mapping progeny. However, since the genetic factor on LG6X2 was associated with a single Axiom marker on the S×SS linkage map, the genetic interval in which it resides was too large to predict likely candidate genes for its control. Further work will be required to functionally validate the role of the candidate genes on LG6b in pelargonidin-3-*O*-acetylglucoside production, as well as to narrow the genetic interval in which the second genetic factor is located on LG6X2 in order to identify suitable candidate genes at this locus.

## Materials and methods

### Plant material

Four cultivars (‘Carisma’ [‘Oso Grande’ × ‘Villanova’], ‘Marlate’ [Sel No 89 258 × 88 0 12], ‘Saga’ [‘Korona’ × ‘Kimberly’], and ‘Senga Sengana’ [‘Sieger’ × ‘Markee’]) were chosen based on the differing content of polyphenolic compounds reported in their fruit by Aaby et al.^10^, and three F_1_ progenies were raised using ‘Senga Sengana’ as the recurrent pollen parent. Three hybrid populations (‘Carisma’ × ‘Senga Sengana’ (C×SS), ‘Marlate’ × ‘Senga Sengana’ (M×SS), and ‘Saga’ × ‘Senga Sengana’ (S×SS)) were obtained through controlled crosses between parental varieties. The resultant seeds were treated with concentrated sulfuric acid for 12 min, rinsed thoroughly in cold water before being germinated in mist chambers (95% relative humidity) at a photoperiod/temperature of 16 h day/20 °C and 8 h night/14 °C. Artificial light was provided by high-pressure sodium lamps (SON/T, 120 µE s^−1^ m^−2^) in periods of low levels of natural light. Parental cultivars and F_1_ plants from each family were propagated from runners and planted in a two-times replicated field experiment, with parental plots replicated up to four times (‘Carisma’ [*n* = 4], ‘Marlate’ [*n* = 3], ‘Saga’ [*n* = 4], and ‘Senga Sengana’ [*n* = 3]), at the experimental site of Graminor Ltd. in Ridabu, Norway in 2015. Each plot contained six plants. Mature fruits from each plot were harvested in the peak season in 2016, flash frozen with liquid nitrogen and stored at −80 °C until analyzed. The number of seedlings in each mapping population were as follows: C×SS (*n* = 48); M×SS (*n* = 47); S×SS (*n* = 45).

### Chemicals used

Quercetin-3-*O*-rhamnosylglucoside (rutin), gallic acid, chlorogenic acid, and ellagic acid were purchased from Sigma Aldrich Ltd. (St. Louis, MO, USA). Pelargonidin-3-*O*-glucoside was purchased from Polyphenols Laboratories AS (Sandnes, Norway). Formic acid (98–100%) and methanol were obtained from Merck KGAa (Darmstadt, Germany). Acetonitrile was sourced from VWR Chemicals (Fontenay-sous-Bois, France). All solvents were of HPLC or analytical grade, and water was of Milli-Q quality (Millipore Corp., Bedford, MA, USA).

### Polyphenolic compound extraction

Fruit samples from the four parental lines and from the progeny of the three mapping populations were partially thawed and homogenized in a food processor (CombiMax 700, Braun GmbH, Kronberg, Germany). Phenolic compounds were extracted from duplicate aliquots (10 g) with methanol (20 ml) by homogenization in a Polytron, PT3100 homogenizer (Kinematica AG, Littau, Switzerland) at 28,000 rpm for 30 s. The extracts were centrifuged at 39,200 × *g* for 10 min at 20 °C (Avanti J-26 XP Centrifuge, Beckman Coulter, USA), following which the supernatants were collected and the insoluble plant material was re-extracted as above with 70% methanol (20 ml). The two pooled supernatants of each sample were combined, and the volume was made up to 50 ml with 70% methanol and stored at −80 °C until analyzed.

### Polyphenolic compound analysis with HPLC-DAD-MS^n^

Extracts were filtered through Millex HV 0.45-μm filters (Merck Millipore Ltd., Cork, Ireland), and analyzed using an Agilent 1100 series HPLC system (Agilent Technologies, Waldbronn, Germany) equipped with an auto-sampler cooled to 4 °C, a diode array detector, and a MSD XCT ion trap mass spectrometer fitted with an electrospray ionization interface as previously described^10^. Chromatographic separation was performed on a Synergi 4-μm MAX RP C12 column (250 mm × 2.0 mm i.d.) equipped with a 5-μm C12 guard column (4.0 mm × 2.0 mm i.d.), both from Phenomenex (Torrance, CA, USA), with one mobile phase consisting of formic acid/water (2/98, v/v) and a second consisting of acetonitrile. Column temperature was 40 °C and injection volume 10 μl. The phenolic compounds were identified based on their UV–vis spectra (220–600 nm), mass spectra and retention times relative to external standards and comparison with previous results^10,47^. The phenolic compounds were classified based on their characteristic UV–vis spectra and quantified by external standards. Anthocyanins were quantified against pelargonidin-3-*O*-glucoside (at 520 nm), flavonols against rutin (at 360 nm), ellagic acid and ellagic acid glycosides against ellagic acid (at 360 nm), and the ellagitannin agrimoniin against gallic acid (at 260 nm). Hydroxy cinnamic acids (HCA) (at 320 nm) and cinnamoyl glucose (at 280 nm) were quantified against chlorogenic acid (at 320 nm). The results were expressed as mg per 100 g of fresh weight (mg 100 g^−1^ FW).

### Phenolic compound quantification for genetic analysis

Means and standard errors for the concentrations of all polyphenol compounds were calculated for each of the parental cultivars, while for the F_1_ progeny the means and standard deviations were calculated. The computations were done with the R function ‘aggregate’^48^. The polyphenol concentrations were scaled using the ‘preProsess’ function in the R package Caret^49^ with the option ‘method = scale’ which divides each observation by the standard deviation of the analyzed compound. A principal component analysis (PCA) was subsequently performed on the scaled data using the prcomp package from core R. Finally, the scores of the two first dimensions in the PCA were plotted with the ggplot package^50^.

### Molecular marker acquisition

DNA was extracted from the parental lines and the progeny of the three mapping populations with the DNeasy Plant Minikit (Qiagen) and quality was determined using a QIAgility spectrophotometer (Qiagen). Samples passing the minimum quality threshold (a 260/280 ratio between 1.8 and 2.0) were normalized to 10 ng μl^−1^ following quantification using a Qubit fluorometer (Thermo Scientific) against known standards. A total of 140 progenies from the three mapping populations (C × SS (*n* = 48); M × SS (*n* = 47); S × SS (*n* = 45)) and the four parental cultivars were genotyped using the Axiom i35K strawberry array (ThermoFisher)^51^ on a GeneTitan instrument (ThermoFisher). Genotyping was done for each individual using the Axiom Analysis Suite software (ThermoFisher) running default quality and SNP-calling parameters.

### Genome-wide association analysis (GWAS)

The segregation data were filtered with snpReady^52^ using a minor allele frequency of 0.05 and markers with more than 5% missing data were excluded from further analysis. Missing data were imputed using the ‘knni’ option in snpReady^53^. All data were combined into a single dataset for subsequent analysis. Since there was no a priori way of assigning all Axiom markers to the subgenomes of the *F.* × *ananassa* ‘Camarosa’ genome sequence^26^ for GWAS analysis, the sequences of all informative iStraw35k array markers were used as queries to interrogate the *F. vesca* v4.0 genome sequence^25^ using a local BLAST database running default parameters. The positions determined for each marker were used for GWAS and subsequently, a subset of markers that were assigned sub-genomic locations by Hardigan et al.^27^ were used to create a Manhattan plot from ‘Camarosa’ genome sequence coordinates, and for the identification of genomic intervals for candidate gene identification. A GWAS was conducted for every polyphenol compound analyzed (Table 1) using GAPIT^54^, implemented in R^48^. The kinship matrix^55^ was calculated using all the useful markers, and five principal components were included as covariates. The primary model was constructed with the general linear model (GLM) algorithm^56^ using a minor allele frequency (MAF) of 0.05. An alternative model was developed using the mixed linear model (MLM)^57^ algorithm with the same covariates as with the GLM. Manhattan plots of the −log_10_(*p*) values were created with the R package qqman^58^ using default settings including the ‘suggestiveline’ (−log_10_(1e−5)) and ‘genomewideline’ (−log_10_(5e−8)) arguments.

### Linkage map construction, QTL analysis, and mapping of qualitative trait loci

Data for each mapping population were considered separately for linkage map construction. The SNP data from the parental genotypes of each progeny were scrutinized initially, and those SNPs that were heterozygous in at least one parent were retained. The remaining monomorphic SNPs, and those for which data from either parent were missing were discarded. The SNP segregation data were retained for further analysis if the progeny contained only genotypes predicted from the parental genotype combination, and for which there were 8% or fewer missing values. Linkage maps were constructed separately for each mapping progeny from all informative markers using JOINMAP 4.1 (Kyazma, NL). Marker placement was determined using regression mapping with a minimum logarithm of odds (LOD) score threshold of 3.0, a recombination fraction threshold of 0.35, a ripple value of 1.0, a jump threshold of 3.0 and a triplet threshold of 5.0. Mapping distances were calculated using the Kosambi mapping function, and homeologous subgenome determination (A, b, X1, and X2) for each linkage group followed the nomenclature of Sargent et al.^59^. The marker data of Hardigan et al.^27^ were used to determine the ‘Camarosa’ chromosome homeologues for each of the identified linkage groups and subsequent BLAST analysis of specific chromosome homeologues was performed to assign a physical position to all mapped markers not placed on the ‘Camarosa’ genome sequence by Hardigan et al.^27^.

QTL analyses were performed separately for the three mapping progenies using interval mapping implemented in MAPQTL 6.0 (Kyazma, NL) with a step size of 1.0 cM, and the percentage phenotypic variance explained and associated LOD values were calculated. The linkage maps and associated LOD plots presented were plotted with MapChart 2.1 using the chart function. Following identification of significant major QTL, some traits were coded as qualitative traits and mapped as discrete genetic loci using JOINMAP 4.1 following the procedure described above. Allele-effect box plots of markers co-segregating in individual populations with qualitative phenotypic trait scores were plotted using the quantitative phenotypes for individuals in all three mapping populations and the genotypes of the most closely associated markers mapped.

### Candidate gene identification and SNP characterization

Initially, the FvH4 v4.0 a2 and ‘Camarosa’ gene predictions within two regions (*Fvb1*: 6–9 Mb and *Fvb6*: 34–36 Mb) of the *F. vesca* v4.0 genome sequence and (*Fvb1-3*: 6.5–9 Mb and *Fvb6-2*: 31–33.5 Mb) of the ‘Camarosa’ genome were downloaded from the Genome Database for Rosaceae. The NCBI_nr, Araport11, Swissprot and TrEMBL annotations of all genes within both intervals were scrutinized (https://www.rosaceae.org/analysis/252)^29^, following which the predicted coding sequences (CDS) were imported into OmicsBox (https://www.biobam.com/omicsbox), and additional information (GO annotations, Interpro information, EggNOG GOs) was added, KEGG pathway information was obtained and candidate genes were identified. Subsequently, synteny comparison between the *F. vesca* and ‘Camarosa’ genomes was performed using the Tripal synteny viewer implemented on the Genome Database for Rosaceae^29^ producing synteny comparisons for all genes within the homologous regions identified. Gene transcript data in *F. vesca* fruits during ripening for the candidate genes identified was retrieved from the strawberry (*F. vesca*) eFP Browser (http://mb3.towson.edu/efp/cgi-bin/efpWeb.cgi)^16^.

## Supplementary information

Figure S1

Figure S2

## Data Availability

If the paper is accepted, data will be made available as supplementaries.
