# Supplementary figures and images for: Major-effect candidate genes identified in cultivated strawberry (Fragaria × ananassa Duch.) for ellagic acid deoxyhexoside and pelargonidin-3-O-malonylglucoside biosynthesis, key polyphenolic compounds
Source: Hortic Res. 2020 Aug 1;7:125. doi: 10.1038/s41438-020-00347-4 (PMC7395118; doi:10.1038/s41438-020-00347-4)

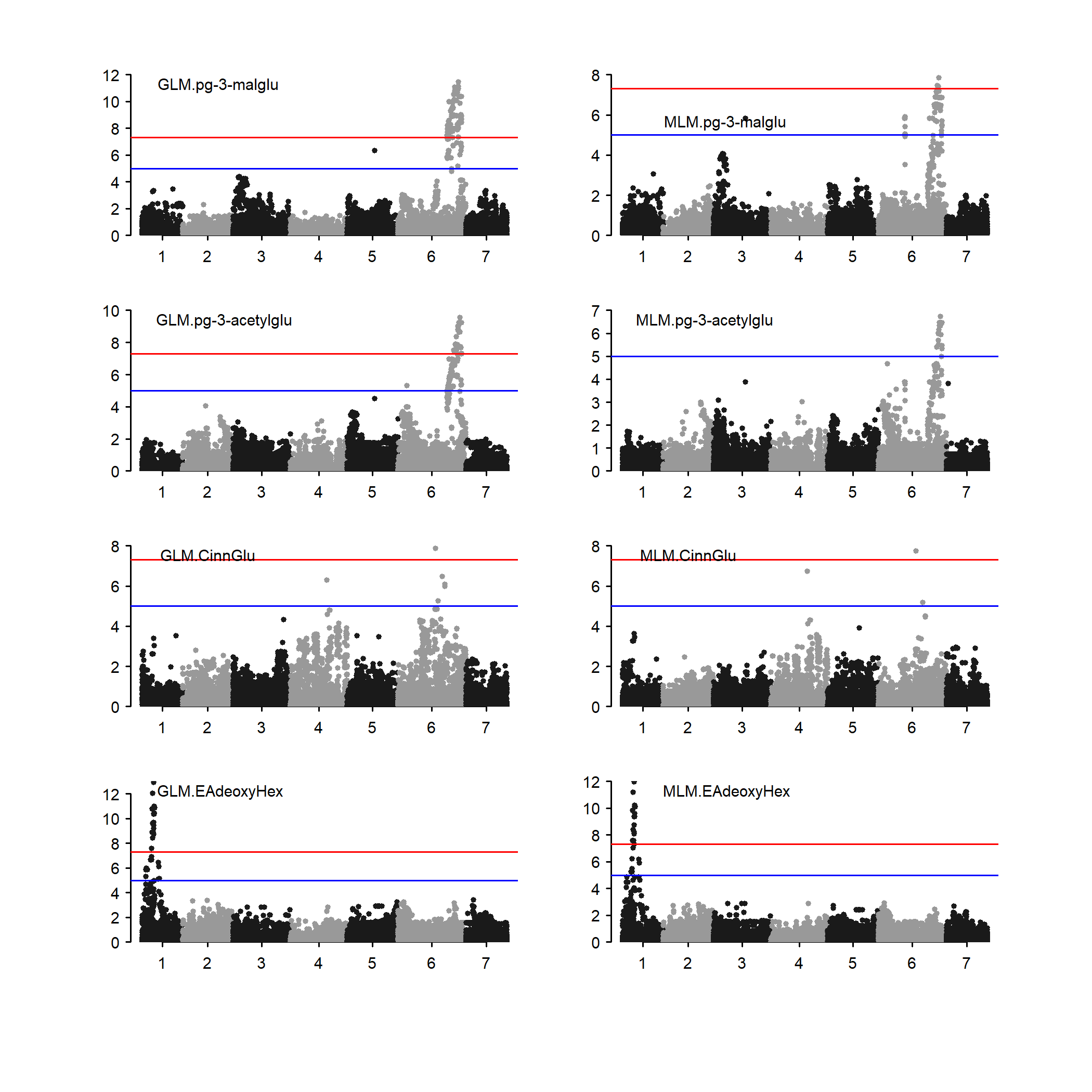

Supplement: Supplementary file 1 — Figure S1 [file 41438_2020_347_MOESM1_ESM.tif]

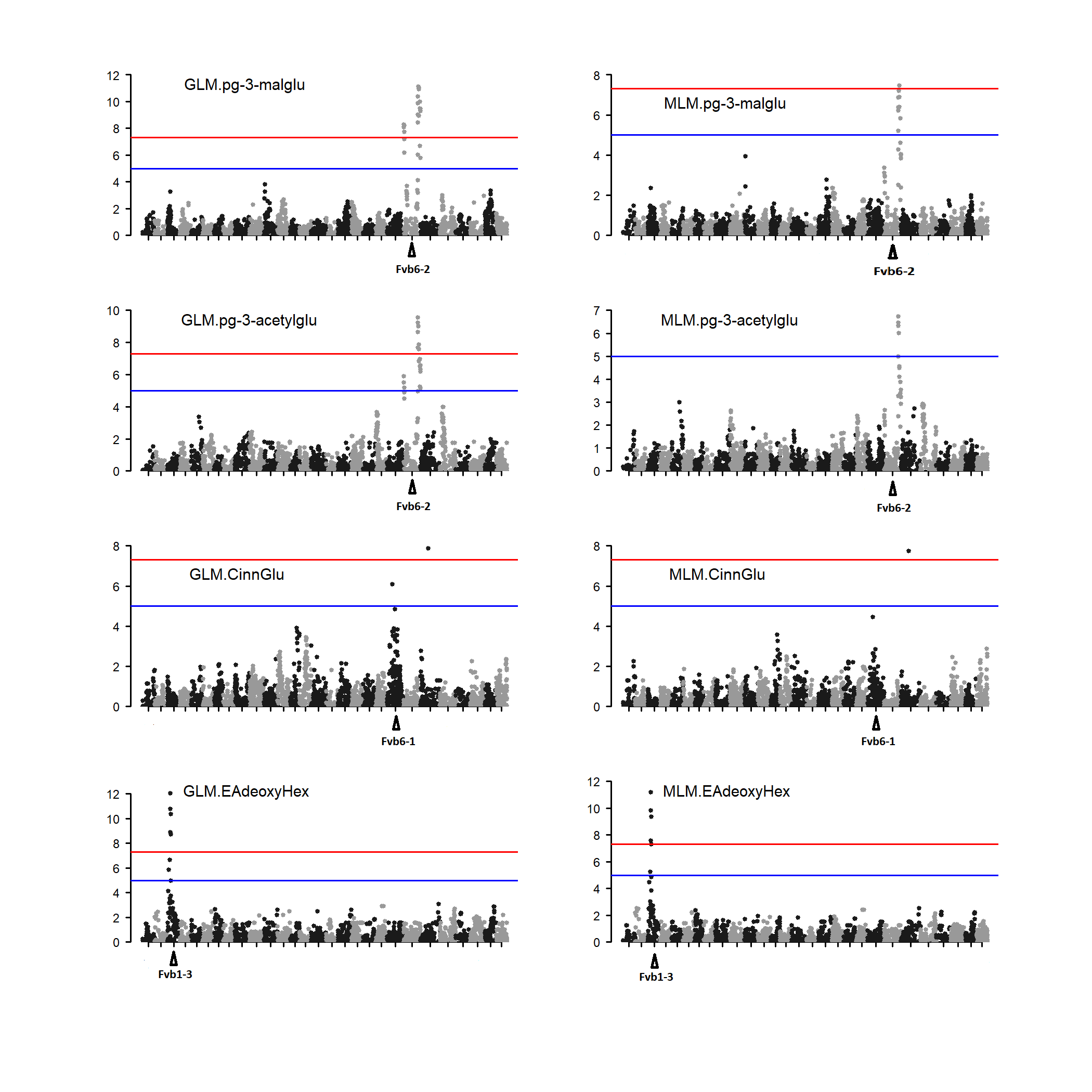

Supplement: Supplementary file 2 — Figure S2 [file 41438_2020_347_MOESM2_ESM.tif]
